# Rapidly growing unilateral pseudoangiomatous stromal hyperplasia in a 10-year-old girl: A case report and review of management strategies

**DOI:** 10.1097/MD.0000000000046548

**Published:** 2025-12-12

**Authors:** Yimei Tang, Xinlin Lv, Xiaorong Han

**Affiliations:** a Department of Thyroid Breast Surgery, Chengdu Women’s and Children’s Central Hospital, School of Medicine, University of Electronic Science and Technology of China, Chengdu, China.

**Keywords:** benign breast disease, PASH, pseudoangiomatous stromal hyperplasia

## Abstract

**Rationale:**

Pseudoangiomatous stromal hyperplasia (PASH) is a benign breast condition characterized by collagen fiber-segmented, blood vessel-like clefts lined by spindle cells, lacking red blood cells. First described by Vuitch et al in 1986, PASH typically presents with benign imaging features and is often identified incidentally during investigations for other breast conditions.

**Patient concerns:**

This case study documents a rare instance of unilateral breast PASH in a 10-year-old girl, which demonstrated rapid growth over a short period, necessitating surgical excision. The patient presented with a rapidly enlarging, painless right breast mass over 2 months. Clinical examination revealed a soft, poorly defined mass measuring approximately 15 × 15 cm with overlying venous engorgement. Imaging studies, including ultrasound and magnetic resonance imaging, showed a low echogenicity mass with irregular borders and non-uniform enhancement.

**Diagnoses:**

Histological features consistent with PASH.

**Interventions:**

Surgical intervention confirmed a well-circumscribed mass with intact capsule.

**Outcomes:**

Postoperative follow-up over 1 year revealed no recurrence.

**Lessons:**

PASH, typically diagnosed incidentally, can exhibit rapid growth and variable imaging characteristics, sometimes mimicking malignant conditions. Its occurrence in children and adolescents is uncommon, with most cases presenting as benign. The association with hormonal factors, observed in adults and those receiving hormonal treatments, suggests a potential link with endocrine changes. In conclusion, while PASH generally has a favorable prognosis, large or rapidly growing lesions in pediatric patients should be managed surgically to prevent potential functional and cosmetic issues.

## 1. Introduction

Pseudoangiomatous stromal hyperplasia (PASH) is defined by the presence of collagen fiber-separated, vessel-like clefts within breast tissue.^[1]^ Despite their resemblance to blood vessels, these structures do not contain red blood cells and are lined with spindle cells rather than endothelial cells. This condition was 1st identified and named by Vuitch et al in 1986.^[2]^ PASH typically shows benign imaging characteristics and is often found incidentally during investigations for other breast disorders.^[3]^ Although cases have become more frequently reported in recent years,^[4–6]^ its occurrence in children and adolescents remains rare. Existing literature suggests a potential association between PASH and hormonal levels,^[7]^ with the condition in younger patients possibly linked to breast development. This article presents a case of a 10-year-old girl, with developed breast tissue, who experienced rapid growth of unilateral PASH, which led to surgical removal.

## 2. Case report

**Figure 1.**
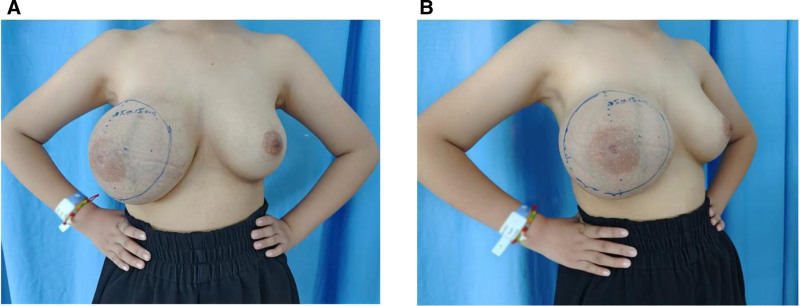
PASH patient before surgery. Physical examination reveals significant enlargement of the right breast compared to the left (A), with noticeable venous dilation on the skin surface (B). A mass approximately 15 × 15 cm is present, with unclear borders. PASH = pseudoangiomatous stromal hyperplasia.

The patient is a 10-year-old girl who presented with a painless mass in the right breast that had been enlarging rapidly for the past 2 months. She had no significant past medical or family history. Menarche occurred at age 9, and breast development on the contralateral side was normal. Physical examination revealed an enlargement of the right breast with overlying venous engorgement. A soft, ill-defined mass approximately 15 × 15 cm in size was palpated (Fig. 1A and B).

**Figure 2.**
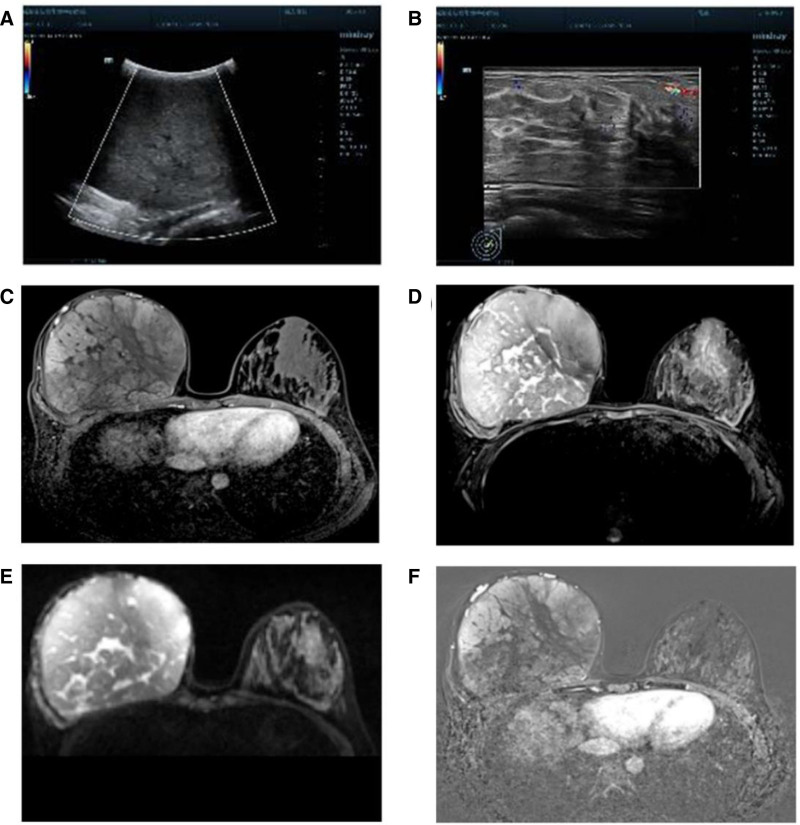
Preoperative ultrasonography (A, B) and MRI (C–F) of bilateral breasts. MRI = magnetic resonance imaging.

Ultrasound of the right breast showed no distinct glandular echoes, but a weakly echogenic mass with a transverse diameter of about 9.3 cm and clear, regular margins was observed (Fig. 2A and B). Magnetic resonance imaging revealed a round mass in the right breast, measuring approximately 12.6 × 11.2 × 13.3 cm, with smooth margins, isointense on T1-weighted imaging (Fig. 2C), slightly hyperintense on fat-saturated T2-weighted imaging (Fig. 2D), and heterogeneous internal signal with linear high signal areas on T2-weighted imaging (Fig. 2E). Diffusion-weighted imaging and apparent diffusion coefficient maps showed isointensity (Fig. 2F). Enhancement on post-contrast images was heterogeneous with a characteristic influx pattern. Subsequent surgical excision of the mass revealed a well-circumscribed tumor with an intact capsule.

**Figure 3.**
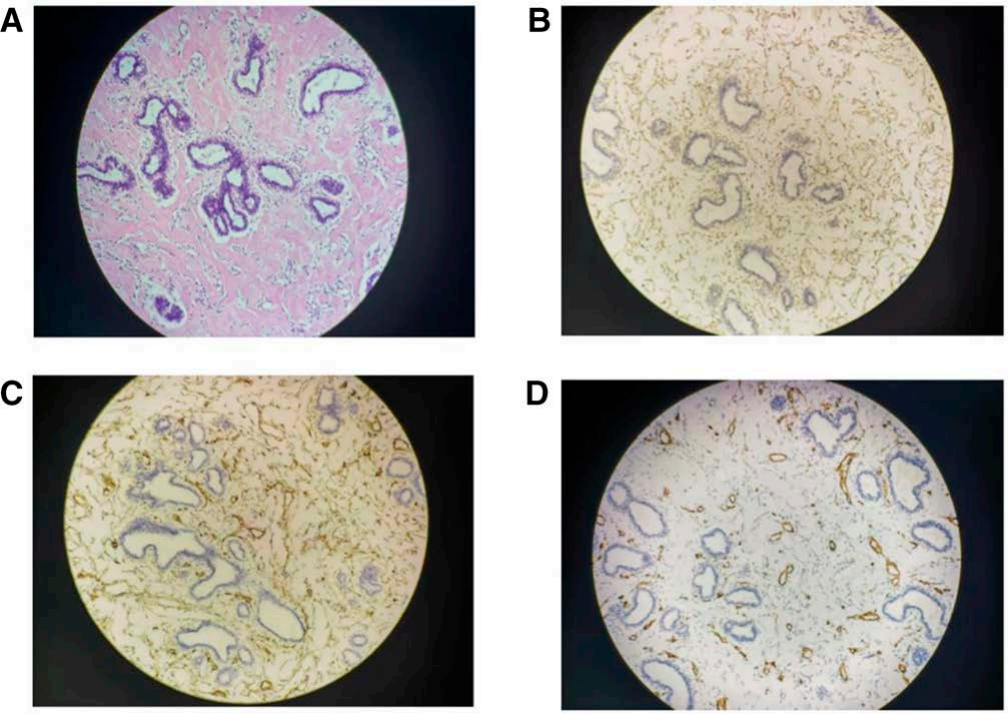
Postoperative of pathological images. In the HE staining, there is a proliferation of myofibroblasts, with irregular fissure-like spaces observed in the dense collagen stroma, without evident nuclear mitotic figures or atypia (A). IHC results indicate that vimentin (B) and CD34 (C) are highly expressed in myofibroblasts, while CD31 is negative (D). IHC = immunohistochemical.

Pathological examination confirmed PASH with stromal proliferation, glassy degeneration, and irregular cleft-like spaces. In the HE staining, there is a proliferation of myofibroblasts, with irregular fissure-like spaces observed in the dense collagen stroma, without evident nuclear mitotic figures or atypia (Fig. 3A). Immunohistochemistry showed positive staining for Vimentin I (Fig. 3B) and CD34 (Fig. 3C), but negative for CD31 (Fig. 3D), CK, Desmin, PR, D2-40, S-100. Ki-67 expression is <1%. Follow-up for 1 year post-surgery showed no signs of recurrence.

## 3. Discussion

PASH is frequently identified incidentally during the assessment of other breast conditions, whether benign or malignant.^[8]^ PASH cases in children and adolescents have also been occasionally reported (Table 1). Clinical reports indicate that PASH lesions can exhibit rapid growth within a short time frame.^[9]^ Typically, PASH presents as a painless, elastic, and mobile mass that is often devoid of a distinct capsule.^[10]^ Ultrasound is the preferred imaging modality for evaluating breast masses in pediatric and adolescent populations.^[11]^ The ultrasonographic appearance of PASH frequently resembles that of fibroadenomas, typically presenting as localized, oval, hypoechoic masses.^[12]^ However, PASH can also manifest as masses with higher echogenicity, poorly defined margins, and increased posterior acoustic enhancement. Cystic components or calcifications are rare, though some PASH lesions may show “pseudo-vascular” structures as linear anechoic areas. While most PASH cases exhibit benign characteristics, some may present with features that mimic malignancy.^[13]^ PASH and virginal breast hypertrophy are similar diseases. Some areas of virginal breast hypertrophy resembled pseudoangiomatous stromal hyperplasia. We discuss the differences between PASH and virginal hypertrophy in Table 2. In the present case,^[14]^ the mass’s soft texture may have led to an initial misinterpretation as a physiological enlargement. The rapid expansion of the mass prompted medical evaluation, as large lesions can significantly impact breast development and cosmetic outcomes.

**Table 1:** Summary of previously reported cases of PASH.

Study	Year	Patient age in years	Size	Management strategies	Recurrence
Singh K A et al	2007	12	22 × 17 cm	Subcutaneous mastectomy with an inferior base pedicle of the nipple–areolar complex.	Recurrence leading to bilateral mastectomy
Baker M et al	2011	10	1.2 × 1.0 cm	Incisional biopsy	No complaint of mass enlargement or of continued associated discomfort at 2-year follow-up.
Abdelrahman T et al	2015	13	35 × 20 cm	Excision with reduction mammoplasty	No recurrence at 18-month follow-up
Inka T et al	2018	16	6 × 3.5 cm	Surgical excision	Not mentioned
Koksal H et al	2020	13	16 × 14 cm	Tru-cut biopsy	No recurrence at 6-month follow-up

PASH = pseudoangiomatous stromal hyperplasia.

**Table 2:** Differential diagnosis between PASH and virginal breast hypertrophy.

Feature	Pseudoangiomatous stromal hyperplasia (PASH)	Virginal breast hypertrophy
Definition	Benign stromal proliferative disorder of the breast, forming pseudoangiomatous structures.	Excessive unilateral/bilateral breast development during puberty or congenitally, often linked to hormonal sensitivity or genetic factors.
Symptoms	A palpable and painless breast mass was found.	Marked breast enlargement (unilateral/bilateral), potential pain, skin tension, or psychological distress.
Growth Pattern	Focal, ill-defined mass; rapid growth may occur (requires exclusion of concurrent lesions).	Diffuse glandular and adipose tissue hyperplasia with uniform enlargement.
Hormonal Association	Intense stromal cell progesterone-receptor (PR) positivity was present in PASH in most cases. Faint stromal nuclear reactivity for estrogen-receptors (ER) was observed.	Associated with heightened hormonal sensitivity during puberty or genetic predisposition.
Palpation Findings	A solitary, firm, painless, well-circumscribed, and freely mobile mass mimicking fibroadenoma. Sometimes, the mass may enlarge rapidly and mimic a malignant tumor.	Soft, homogeneous glandular hyperplasia without focal nodules or hard masses.
Ultrasound	Further assessment with ultrasonography typically reveals an oval, irregular, hypoechoic, or mixed echogenic mass without posterior acoustic enhancement or shadowing.	Uniform glandular thickening, isoechoic to normal breast tissue; no focal masses or abnormal vascularity.
Mammography	The mammographic findings consist of a well-circumscribed, round to oval density without calcification.	Dense glandular tissue with uniform distribution; no focal density abnormalities or architectural distortion.
MRI	T2-weighted hyperintensity with strong enhancement; reticular septations or slit-like spaces.	Homogeneous glandular enhancement; no focal masses or abnormal enhancement.

PASH is predominantly observed in premenopausal women and postmenopausal women undergoing hormone replacement therapy.^[15]^ Additionally, it has been documented in transgender men receiving hormone therapy and patients on polypharmacy for neurological conditions, including clonazepam, valproate, and risperidone, which may elevate progesterone levels and potentially stimulate PASH growth.^[12]^ Existing literature suggests a correlation between PASH and hormonal factors, though the precise etiology remains unsubstantiated by direct research.

Breast pathologies are relatively rare in children and adolescents compared to adults.^[16]^ Among all adult breast lesions, similar conditions can occur in younger individuals. Benign tumors, such as fibroadenomas, predominate in this demographic, comprising 80% to 95% of cases, while malignant lesions are infrequent, accounting for proximately 0.02% and often include metastatic tumors such as lymphoma, rhabdomyosarcoma, and neuroblastoma.^[17,18]^ The median age for breast tumors in the pediatric and adolescent population is around 17 years, making the occurrence of breast tumors in children under 10 years particularly rare.^[19]^

Diagnosis of PASH is typically achieved through core needle biopsy or surgical excision with subsequent pathological and immunohistochemical analysis.^[20]^ Differential diagnoses include fibroepithelial tumors and vascular lesions such as angiosarcoma.^[21]^ Overall, PASH is associated with a favorable prognosis, with most cases exhibiting no significant malignancy.^[22]^ PASH lesions usually demonstrate slow or no growth and may be multifocal. While observation is appropriate for many cases, those with rapid growth, such as the case presented here, may necessitate surgical intervention.^[23]^ Follow-up imaging for 1 year post-surgery showed no recurrence. In some instances, extensive interventions like mastectomy or reductive mammoplasty have been performed due to the size or diffuse nature of the lesions. Tamoxifen has been reported to cause regression in some PASH cases, though the efficacy remains under investigation.^[24]^ PASH can recur, with reported local recurrence rates of 15% to 22%,^[25]^ thus necessitating close monitoring or surgical intervention for lesions larger than 3 cm or those demonstrating progressive growth.

## Author contributions

**Investigation:** Xinlin Lv.

**Writing – original draft:** Yimei Tang.

**Writing – review & editing:** Xiaorong Han.
